# Ether Phosphatidylserine from Soft Coral *Sclerophytum heterospiculatum* Reveals Antioxidant Activity and Modulates Lipid Composition in LPS-Activated Human Microglial HMC-3 Cells

**DOI:** 10.3390/md24060188

**Published:** 2026-05-23

**Authors:** Elena T. Bizikashvili, Arina I. Ponomarenko, Ekaterina V. Ermolenko, Igor V. Manzhulo

**Affiliations:** A.V. Zhirmunsky National Scientific Center of Marine Biology, Far Eastern Branch, Russian Academy of Sciences, ul. Palchevskogo 17, 690041 Vladivostok, Russia; arina.ponomarenko.93@mail.ru (A.I.P.); ecrire_711@mail.ru (E.V.E.); i-manzhulo@bk.ru (I.V.M.)

**Keywords:** ether phosphatidylserine, microglia, oxidative stress, lipidomics, soft coral

## Abstract

Ether phospholipids from marine organisms represent an understudied class of bioactive lipids with unique structural features. In this study, we isolated, for the first time, an ether phosphatidylserine (ePS) species from the soft coral *Sclerophytum heterospiculatum* and assessed its biological activity on human microglial clone 3 (HMC-3) cells. The isolated ePS contained an ether bond at the sn-1 position and very-long-chain polyunsaturated fatty acids (PUFA) (24:5) at the sn-2 position. Using an MTS assay, we demonstrated that ePS was non-cytotoxic at all tested concentrations (0.39–100 μg/mL) and even increased microglial proliferation at 50–100 μg/mL. In microglial cells activated by lipopolysaccharide (LPS-activated), ePS significantly reduced production of reactive oxygen species (ROS), nitric oxide (NO), and malondialdehyde (MDA). A lipidomic analysis by HPLC–MS/MS revealed that ePS modulated the membrane lipid composition of microglial cells, increasing the content of polyunsaturated phosphatidylserines (PS 36:3, PS 40:5) and decreasing the levels of phosphatidylinositols (PI 18:1/20:4; PI 18:0/20:4, 18:1/20:3). Furthermore, a fatty acid analysis showed that ePS prevented LPS-induced accumulation of saturated fatty acids and preserved PUFA levels in HMC-3 cells. These findings suggest that marine-derived ePS can be considered as promising agents with antioxidant and anti-inflammatory properties.

## 1. Introduction

Investigation of marine natural products has gained significant momentum in recent decades due to the high structural diversity of compounds synthesized by marine organisms. Soft corals (Cnidaria: Anthozoa: Octocorallia) have been considered the second-most common group of macrobenthic animals after reef-building corals [1]. Octocorals of the genus Sclerophytum (formerly Sinularia) contain a vast range of biologically active substances with anti-inflammatory [2,3], antimicrobial [4], cytotoxic [4,5], antimalarial [6], and antidiabetic effects [7]. The biological potential of octocorals has been largely attributed to secondary metabolites such as terpenes and steroidal glycosides, leaving the bioactivity of their lipidome largely unexplored. Octocorals of the genus Sclerophytum are rich in ether lipids, whose content makes up to 90% of total phospholipids (PL) [8,9]. However, individual lipid species, in particular polar lipids such as phosphatidylserine (PS), have not been isolated, and their specific biological properties remain completely unknown. Thus, *Sclerophytum heterospiculatum*, a typical commonly distributed representative of the genus, may represent an underexplored source of bioactive ether lipids.

Ether lipids are a unique class of PL that have an alkyl chain attached to the sn-1 position via an ether bond in glycerol backbone [10]. Ether lipids are chemically distinct from their diacyl counterparts and, therefore, impart unique structural characteristics to biological membranes that affect such properties as membrane fluidity and membrane fusion [10]. PS is a critical PL in cellular membranes, with high concentrations found in the brain [11]. It plays a key role in the development and maintenance of cell membranes in the central nervous system (CNS), facilitating intercellular communication, receptor activation, and nutrient and waste transport across cellular membranes [12]. A characteristic feature of ether phosphatidylserines (ePS) from *S. heterospiculatum* is the presence of tetracosapolyenoic fatty acids (TPA, 24:5 and 24:6) at the *sn-2* position [13]. This combination of an ether bond at the *sn-1* position and very-long-chain polyunsaturated fatty acids (PUFA) at the *sn-2* position results in a unique molecular architecture. The biological activity and, specifically, the antioxidant potential of such unique ePS molecules have never been investigated.

One of the main cellular models for studying the anti-inflammatory and neuroprotective potential of new compounds is the microglial cell. Microglial cells are unique myeloid cells residing in the parenchyma of the healthy CNS [14]. As resident immune cells of the CNS, microglia are the primary source of ROS and proinflammatory cytokines in neuroinflammatory and neurodegenerative conditions such as those in Alzheimer’s and Parkinson’s diseases [14]. Testing isolated ePS on human microglial cells will make it possible to assess its activity in a cellular system that is directly implicated in CNS pathologies where oxidative stress plays a key role.

The aim of this study was to isolate specific ether phosphatidylserine (ePS) species containing an alkyl ether bond at the *sn-1* position and very-long-chain polyunsaturated fatty acid (24:5) at the *sn-2* position from the soft coral *Sclerophytum heterospiculatum*, and to evaluate its cytotoxic and antioxidant activity in LPS-activated human microglial cell line (HMC-3), as well as its modulatory effects on membrane lipid composition.

## 2. Results

### 2.1. Isolation and Characterzation of ePS from Sclerophytum heterospiculatum

Column chromatography on silica gel of the total lipid extract from *S. heterospiculatum* yielded 20 fractions, which were analyzed by thin-layer chromatography (TLC) (Figures S8 and S9). Fraction 10 co-migrated with phosphatidylserine standard (PS 18:0/18:1) and was clear; therefore, it was collected as the ePS fraction. HPLC-MS/MS analysis of fraction 10 revealed a major molecular ion at *m*/*z* [M-H]^-^ 850.596, corresponding to an ether-linked phosphatidylserine (ePS) with an alkyl chain at the *sn-1* position (18:0e) and a very-long-chain polyunsaturated fatty acid (24:5) at *sn-2* position (Figure S7). Thus, the isolated ePS fraction consists predominantly of 1-*O*-stearoyl-2-tetracosapentaenoyl-*sn*-glycero-3-phosphoserine. In addition to isolated ePS, in this study, we examined the effect of the total phospholipid fraction (extPL) from *S. heterospiculatum* on HMC-3 microglial cells. The aim of this analysis was to determine whether the phospholipid complex exerts a cytotoxic or antioxidant effect on microglia, as well as to identify potential novel effects that may arise due to the presence of multiple compounds in the extPL.

### 2.2. Biological Activity Assay

#### 2.2.1. Cytotoxicity of ePS and extPL Towards Microglial Cells

**Figure 1:**
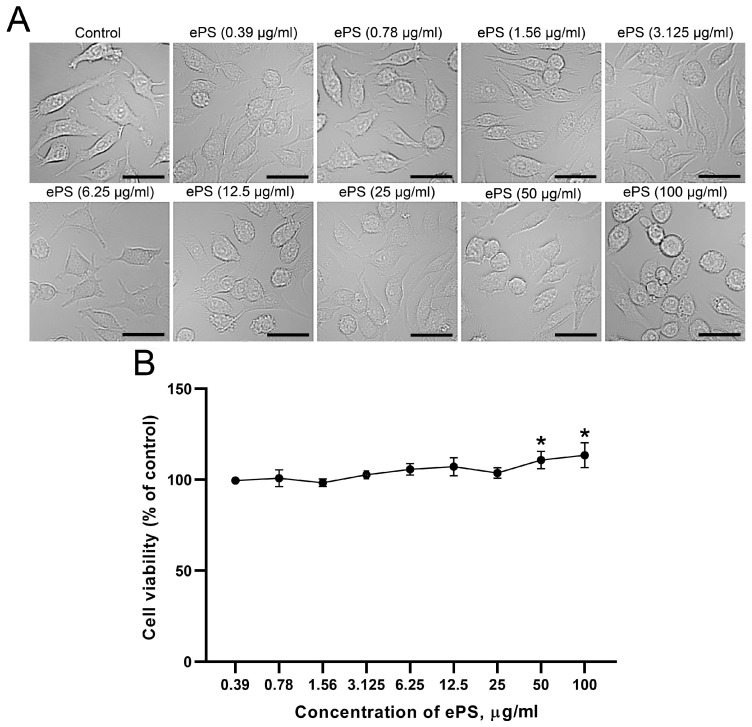
Cytotoxicity of ePS (ether phosphatidylserine isolated from *Sclerophytum heterospiculatum*) on HMC-3 human microglial cells. (**A**) Images of HMC-3 cells exposed to ePS at different concentrations (0.39–100 µg/mL) for 72 h. Scale bar: 25 µm. (**B**) Cell viability of HMC-3 cells exposed to ePS at different concentrations (0.39–100 µg/mL) as inferred by the MTS assay. Data are presented as mean ± SEM; *n* = 18 (number of biological repeats). * *p* < 0.05 (Student’s *t*-test) Abbreviations: ePS 0.39–100 µg/mL—cells treated with ePS at indicated concentrations.

In this study, we assessed the effect of ePS and extPL on the viability, morphology, and proliferation of human microglial cells. The lack of cytotoxicity of the tested compounds on HMC-3 cells was confirmed by MTS assay with 72 h exposure of cells. Micrographs of the HMC-3 culture also showed no pronounced morphological changes indicative of apoptosis or necrosis (Figure 1A). At all concentrations, ePS showed no cytotoxic effect on human microglial cells. The treated cells, like those in the control group, adhered well to the plastic surface in the culture, exhibited spindle-shaped and rounded morphology, and had homogeneous cytoplasm without inclusions and a large, rounded nucleus with one or two nucleoli. At concentrations of 50 and 100 μg/mL, ePS, vice versa, significantly increased the reduction of tetrazole in human microglial cells, which suggested increased cell proliferation or increased MTS reduction at the cell surface compared to the control group (Figure 1B).

**Figure 2:**
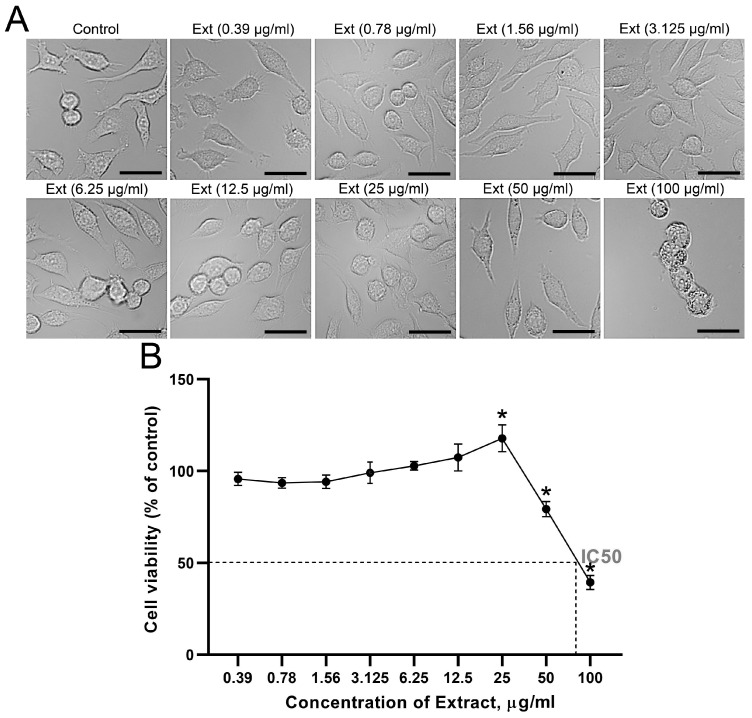
Cytotoxicity of extPL (total phospholipid extract isolated from *Sclerophytum heterospiculatum*) on HMC-3 human microglial cells. (**A**) Images of HMC-3 cells exposed to extPL at different concentrations (0.39–100 µg/mL) for 72 h. Scale bar: 25 µm. (**B**) Cell viability of HMC-3 cells exposed to extPL at different concentrations (0.39–100 µg/mL) as inferred by the MTS assay. Data are presented as mean ± SEM; *n* = 18 (number of biological repeats). * *p* < 0.05 (Student’s *t*-test) Abbreviations: extPL 0.39–100 µg/mL—cells treated with extPL at indicated concentrations.

Unlike ePS, the extPL exhibited a different effect. At concentrations of 0.39 to 12 μg/mL, it showed no cytotoxic effect, while at 25 μg/mL, it significantly increased HMC-3 cell proliferation. At concentrations of 50 and 100 μg/mL, it exhibited a cytotoxic effect on microglial cells (Figure 2B). When a maximum concentration of extPL was used, the cell morphology changed, and signs of apoptosis and cell death appeared: blebbing, loss of adhesive properties, destruction of the nucleus, and vacuolization of cytoplasm (Figure 2A).

#### 2.2.2. Antioxidant Activity of ePS and extPL Towards Microglial Cells

**Figure 3:**
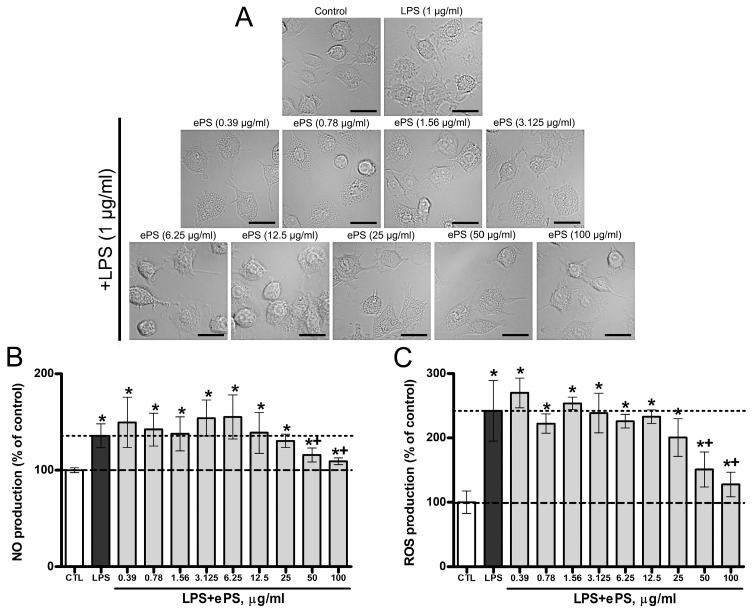
Antioxidant activity of ePS (ether phosphatidylserine isolated from *Sclerophytum heterospiculatum*) against LPS-induced NO and ROS production in HMC-3 cells. Scale bar: 25 µm. (**A**) Morphological changes in HMC-3 cells exposed to LPS (1 µg/mL) and ePS at different concentrations (0.39–100 µg/mL). (**B**) Antioxidant activity of ePS against LPS induced NO production in HMC-3 cells. (**C**) Antioxidant activity of ePS against LPS induced ROS production in HMC-3 cells. Dashed lines represent the control and LPS level for reference. Data are presented as mean ± SEM; *n* = 18 (number of biological repeats). * *p* < 0.05 vs. CTL (control group); + *p* < 0.05 vs. LPS (Student’s *t*-test). Abbreviations: CTL—untreated control cells; LPS—cells treated with lipopolysaccharide (1 µg/mL); ePS 0.39–100 µg/mL—cells treated with ePS at indicated concentrations and LPS (1 µg/mL); NO—nitric oxide; ROS—reactive oxygen species.

After the addition of LPS, there was LPS-mediated activation of microglial cells, expressed as an increase in their size, flattening, formation of a greater number of processes, and vacuolization of cytoplasm. Moreover, cells pre-treated with the maximum concentrations (50 and 100 µg/mL) of ePS partially restored the morphology altered under the effect of LPS (Figure 3A).

ePS prevented the LPS-mediated increase in NO production at concentrations of 50 and 100 μg/mL (133.229 ± 14.428 for LPS vs. 115.784 ± 7.186 for LPS + ePS 50 μg/mL, *p* ˂ 0.05) (Figure 3B) and (133.229 ± 14.428 for LPS vs. 109.207 ± 3.504 for LPS + ePS 100 μg/mL, *p* ˂ 0.05) (Figure 3B). At the same concentrations, ePS reduced the level of ROS in LPS-activated HMC-3 cells (263.957 ± 53.963 for LPS vs. 151.048 ± 27.069 for LPS + ePS 50 μg/mL, *p* ˂ 0.05; 263.957 ± 53.963 for LPS vs. 127.698 ± 19.276 for LPS + ePS 100 μg/mL, *p* ˂ 0.05) (Figure 3C).

**Figure 4:**
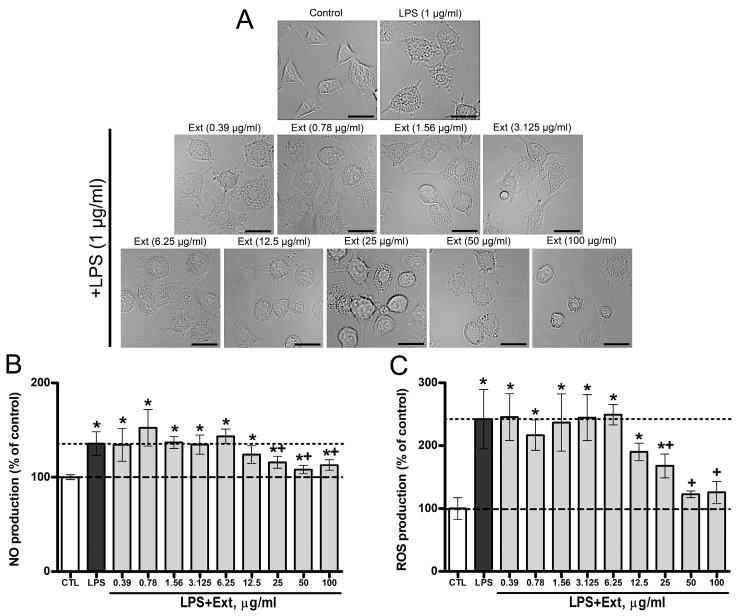
Antioxidant activity of extPL (total phospholipid extract isolated from *Sclerophytum heterospiculatum*) against LPS-induced NO and ROS production in HMC-3 cells. Scale bar: 25 µm. (**A**) Morphological changes in HMC-3 cells exposed to LPS (1 µg/mL) and extPL at different concentrations (0.39–100 µg/mL). (**B**) Antioxidant activity of extPL against LPS induced NO production in HMC-3 cells. (**C**) Antioxidant activity of extPL against LPS induced ROS production in HMC-3 cells. Dashed lines represent the control and LPS level for reference. Data are presented as mean ± SEM; *n* = 18 (number of biological repeats). * *p* < 0.05 vs. CTL (control group); + *p* < 0.05 vs. LPS (Student’s *t*-test). Abbreviations: CTL—untreated control cells; LPS—cells treated with lipopolysaccharide (1 µg/mL); extPL 0.39–100 µg/mL—cells treated with extPL at indicated concentrations and LPS (1 µg/mL); NO—nitric oxide; ROS—reactive oxygen species.

To study the effect of extPL treatment on microglial cells, we used an LPS-mediated inflammation model. Upon addition of extPL to cells incubated with LPS at various concentrations, the effect of extPL on the levels of ROS and NO was observed. extPL reduced the level of NO production when incubated with LPS at concentrations of 25, 50, and 100 μg/mL (138.087 ± 11.601 for LPS vs. 115.89 ± 8.86 for LPS + extPL 25 μg/mL, *p* ˂ 0.05; 138.087 ± 11.601 for LPS vs. 108.112 ± 4.31 for LPS + extPL 50 μg/mL, *p* ˂ 0.05; 138.087 ± 11.601 for LPS vs. 112.984 ± 5.778 for LPS + extPL 100 μg/mL, *p* ˂ 0.05) (Figure 4B). At the same concentrations, extPL decreased the level of ROS in LPS-activated HMC-3 cells (220.509 ± 31.392 for LPS vs. 167.837 ± 18.909 for LPS + extPL 25 μg/mL, *p* ˂ 0.05; 220.509 ± 31.392 for LPS vs. 122.619 ± 5.339 for LPS + extPL 50 μg/mL, *p* ˂ 0.05; 220.509 ± 31.392 for LPS vs. 125.515 ± 17.698 for LPS + extPL 100 μg/mL, *p* ˂ 0.05) (Figure 4C).

**Figure 5:**
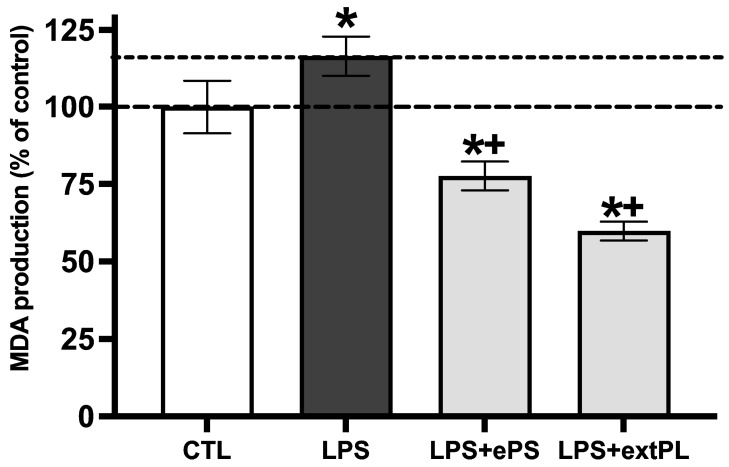
Analysis of MDA production in HMC-3 cell culture exposed to LPS (1 µg/mL), ePS (25 µg/mL), and extPL (25 µg/mL). Dashed lines represent the control and LPS level for reference. Data are presented as mean ± SEM; *n* = 6 (number of biological repeats); * *p* ˂ 0.05 vs. CTL; + *p* ˂ 0.05 vs. LPS (Student’s *t*-test). Abbreviations: CTL—untreated control cells; LPS—cells treated with lipopolysaccharide (1 µg/mL); LPS + ePS—ePS pretreatment (25 µg/mL) + LPS (1 µg/mL); LPS + extPL—extPL pretreatment (25 µg/mL) + LPS (1 µg/mL).

We also attempted to elucidate how ePS and extPL influence MDA formation in microglial cells. extPL reduced MDA levels by half at a concentration of 25 μg/mL when incubated with LPS for 24 h (119.358 ± 4.716 for LPS vs. 58.91 ± 0.677 for LPS + extPL 25 μg/mL, *p* ˂ 0.05) (Figure 5, Table S2). However, ePS reduced MDA levels at a concentration of 25 μg/mL only 1.5-fold (119.358 ± 4.716 for LPS vs. 76.275± 4.428 for LPS + ePS 25 μg/mL, *p* ˂ 0.05) (Figure 5, Table S2).

### 2.3. Molecular Profile of Membrane Lipids of HMC-3 Cell Culture

**Figure 6:**
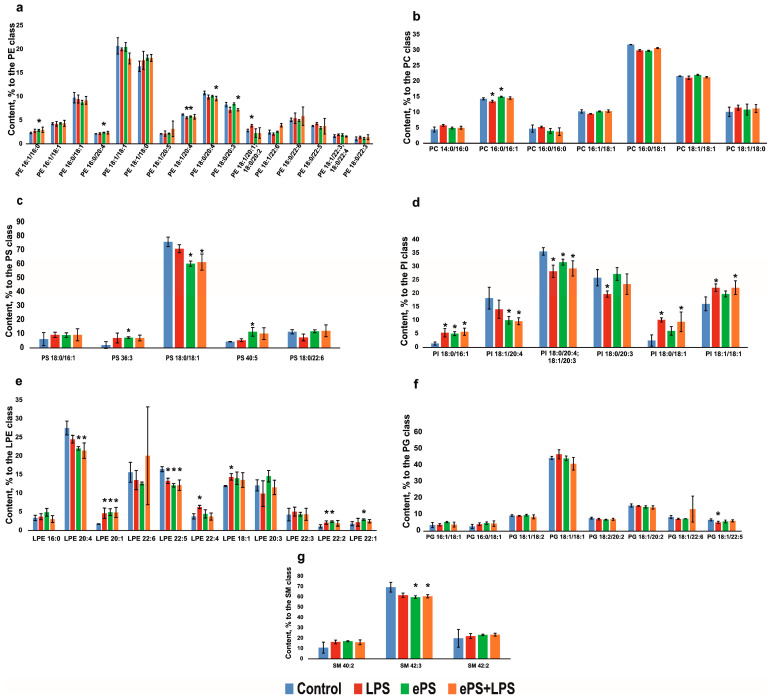
Content of molecular species of phospholipids (PL) in HMC-3 human microglial after 72 h of treatment. Cells were exposed to: Control (Blue bars), LPS (1 µg/mL) (Red bars), ePS (25 µg/mL) (Green bars), ePS + LPS (Orange bars). (**a**) Phosphatidylethanolamines (PE) molecular species; (**b**) phosphatidylcholines (PC) molecular species; (**c**) phosphatidylserines (PS) molecular species; (**d**) phosphatidylinositols (PI) molecular species; (**e**) lysophosphatidylethanolamines (LPE) molecular species; (**f**) phosphatidylglycerols (PG) molecular species; and (**g**) sphingomyelins (SM) molecular species. Molecular species of PL were analyzed by HPLC–MS/MS. Each row represents a distinct molecular species of PL. Data are presented as mean ± SD; *n* = 3 (number of samples analyzed); * *p* ˂ 0.05 (one-way ANOVA by post hoc Tuckey’s HSD test). Abbreviations: Control—untreated control cells; LPS—cells treated with lipopolysaccharide (1 µg/mL); ePS—cells treated with only ePS (25 µg/mL); ePS + LPS—ePS pretreatment (25 µg/mL) + LPS (1 µg/mL).

The total lipid extract of HMC-3 cells contained the following PL: PC, PE, PS, PG, SM, PI, and LPE. Using HPLC–MS/MS, we characterized the molecular profiles of membrane lipids in the HMC-3 cell culture. A total of 60 PL molecular species were identified, including 17 molecular species of PE, 9 of PC, 5 of PS, 8 of PG, 3 of SM, 6 of PI, and 12 of LPE (Figure 6). The major molecular species of PE in the control group were PE 18:1/18:1, PE 18:1/18:0, and PE 18:0/20:4 whose contents were 20.6, 16.3, and 10.7%, respectively (Figure 6a, Table S3). Compared to the control group, in the LPS group, the level of the molecular species PE 18:1/20:4 significantly decreased from 6.1 to 5.4%, whereas the molecular species PE 18:1/20:1; 18:0/20:2 significantly increased from 2.8 to 3.8% (Figure 6a). In the ePS group, the level of molecular species PE 16:1/16:0 significantly increased from 2.2 to 2.8%, PE 16:0/20:4 from 2.1 to 2.3% and that of PE 18:1/20:4 significantly decreased from 6.1 to 5.7% compared to the control group (Figure 6a). In the ePS + LPS group, the molecular species PE 18:0/20:4 and PE 18:0/20:3 significantly decreased compared to the control group, from 10.7 to 9.5% and from 8.2 to 7.2%, respectively (Figure 6a).

In the control group, the major molecular species of PC were PC 16:0/18:1, PC 18:1/18:1, PC 16:0/16:1, and PC 18:1/18:0 whose contents were 31.7, 21.6, 14.3, and 10.0%, respectively (Figure 6b, Table S3). The level of the molecular species PC 16:0/16:1 significantly decreased in the LPS group from 14.3 to 13.4%, but in the ePS group, the level of this molecular species significantly increased from 14.3 to −14.9% compared to the control group (Figure 6b). In the control group, the major molecular species of PG were PG 18:1/18:1, PG 18:1/20:2, and PG 18:1/18:2, whose contents were 44.7, 15.6, and 9.5%, respectively (Figure 6f). The molecular species of PG 18:0/22:5 in the LPS group significantly decreased compared to the control group, from 6.8 to 5.3% (Figure 6f).

The major molecular species of PS in the control group were PS 18:0/18:1, PS 18:0/22:6, and PS 18:0/16:1 whose contents were 75.9, 11.4, and 6.2%, respectively (Figure 6c, Table S3). In the LPS group, no significant changes in the molecular species of PS were observed compared to the control group. Significant changes were found only in the groups treated with ePS. In the ePS group, the levels of PS 36:3 significantly increased from 2.01 to 7.31%, PS 40:5 from 4.37 to 11.45%, and PS 18:0/18:1 significantly decreased from 75.92 to 60.33% compared to the control group (Figure 6c). Also, in the ePS + LPS group, the level of PS 18:0/18:1 significantly decreased from 75.92 to 61.41% compared to the control group (Figure 6c).

In the control group, some of the major PI molecular species were PI 18:0/20:4; 18:1/20:3, PI 18:0/20:3, PI 18:1/18:1, and PI 18:1/20:4 whose contents were 35.7, 25.8, 16.1, and 18.3%, respectively (Figure 6d, Table S3). In the LPS group, the levels of PI molecular species with PUFA significantly decreased, whereas the level of PI molecular species with monounsaturated fatty acids (MUFA) significantly increased. The same pattern was observed in the ePS + LPS group (Figure 6d). For LPE, the major molecular species in the control group were LPE 20:4, LPE 22:6, LPE 22:5, LPE 18:1, and LPE 20:3, with percentages of 27.5, 15.6, 16.4, 11.9, and 12.1%, respectively (Figure 6e). Compared to the control group, in the LPS, ePS, and ePS + LPS groups, there was a significant increase in LPE (20:1 of 4.6, 4.8, and 4.9%, respectively) and a significant decrease in the molecular species of LPE 22:5 (13.3, 12.1, and 12.1%, respectively) (Figure 6e). Only in the LPS group, the molecular species of LPE 22:4 (6.2%) and LPE 18:1 (14.4%) increased significantly compared to the control group (Figure 6e). In the ePS and ePS + LPS groups, the level of the molecular species LPE 20:4 significantly decreased by 22.0 and 21.4%, respectively (Figure 6e). In the LPS and ePS groups, the molecular species LPE 22:2 increased significantly compared to the control group by 2.1 and 2.3%, respectively (Figure 6e).

In total, three molecular species of sphingomyelins (SM) were detected, namely SM 40:2, SM 42:3, and SM 42:2, the contents of which in the control group were 10.7, 69.3, and 19.8%, respectively (Figure 6g).

As shown in Table 1, stimulation of HMC-3 cells with LPS led to a significant increase in the proportion of saturated fatty acids (SFA) in the total lipid composition. The total SFA content increased from 61.288 ± 8.11% in the control group to 81.899 ± 9.95% in the LPS group (*p* ˂ 0.05). This increase was preliminary driven by the accumulation of palmitic (16:0) and stearic (18:0) acids (Table 1). Concurrently, a decrease in the levels of monounsaturated (MUFA) and polyunsaturated (PUFA) fatty acids was observed, but without significant differences. Importantly, pretreatment of cells with ePS (25 µg/mL, 72 h) effectively prevented this proinflammatory shift in the fatty acid profile. In the ePS + LPS group, the total SFA content was 70.42 ± 6.87%, which was significantly lower than in the LPS group and did not differ from control values. Notably, treatment with ePS alone did not alter SFA levels compared to the control group (58.015 ± 8.41%), ruling out a direct effect of ePS on fatty acid metabolism in the absence of an inflammatory stimulus.

### 2.4. Effect of ePS on Microglial Cell Membrane Lipids

**Figure 7:**
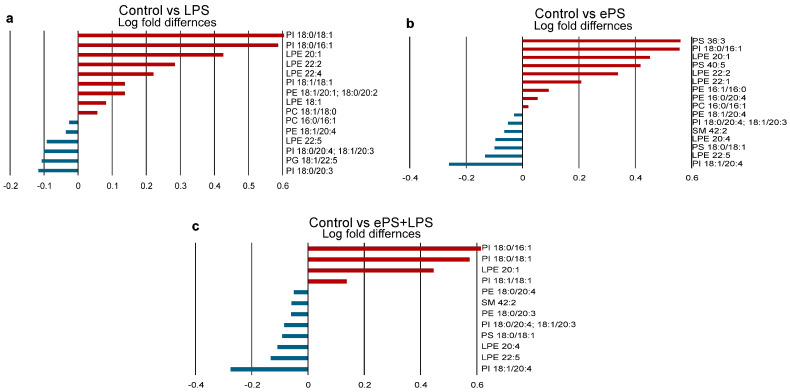
Significant changes in the molecular profile of phospholipids (PL) in HMC-3 human microglial cell under different treatments. HMC-3 cells were treated with (**a**) LPS vs. Control (1 µg/m, 72 h), (**b**) ePS (25 µg/mL, 72 h) vs. Control, or (**c**) ePS + LPS (pretreatment with ePS 25 µg/mL, followed by LPS 1 µg/mL, 72 h) vs. Control. Molecular species of PL were analyzed by HPLC–MS/MS and are presented as log10 (experimental group/control group). Each row represents a distinct molecular species of phosphatidylinositols (PI), phosphatidylglycerols (PG), lysophosphatidylethanolamines (LPE), phosphatidylethanolamines (PE), phosphatidylcholines (PC), phosphatidylserines (PS), and sphingomyelins (SM). The color indicates the magnitude of change (blue is downregulation, and red is upregulation). Data are presented as mean log_2_(FC); *n* = 3 (number of samples analyzed).

To evaluate the effect of ePS isolated from *S. heterospiculatum* on the lipid composition of HMC-3 cell membranes, we carried out a comparative lipidomic analysis of human microglial cells under three experimental conditions: (a) LPS stimulation (1 μg/mL, 72 h); (b) ePS treatment (25 μg/mL) followed by LPS stimulation (ePS + LPS); and (c) ePS treatment only (25 μg/mL, 72 h) (Figure 7).

The stimulation of HMC-3 cells with LPS induced bidirectional changes in the molecular profile of PL (Figure 7a). The pronounced effect was a strong upregulation of several PI molecular species: levels of PI 18:0/18:1 and PI 18:0/16:1 increased compared to the control group (log10(LPS/control) = 0.60 and 0.59, respectively). LPE levels were also elevated, with LPE 20:1 showing an increase (log10 = 0.43), followed by LPE 22:2, LPE 22:4, and LPE 18:1 (log10 ranging from 0.28 to 0.08). In contrast, the level of a distinct set of PL decreased upon LPS treatment. The largest decreases were observed for PI 18:0/20:3 (log10 = −0.12), PG 18:1/22:5 (log10 = −0.11), and PI 18:0/20:4, PI 18:1/20:3 (log10 = −0.10). Moderate decreases were also detected for LPE 22:5 (log10 = −0.09) and PE 18:1/20:4 (log10 = −0.04), while PC 16:0/16:1 showed a minor decrease (log10 = −0.03).

The treatment with ePS modulated the LPS-induced lipid changes (Figure 7b). Levels of several PL that were upregulated by LPS alone remained elevated in the ePS + LPS group: PI 18:0/16:1 and PI 18:0/18:1 showed a persistent increase (log10(ePS + LPS/control) = 0.61 and 0.57, respectively), and LPE 20:1 remained above control levels (log10 = 0.45). However, the ePS treatment also induced distinct changes not observed with LPS alone. Most notably, level of PI 18:1/20:4 was substantially reduced compared to the control group (log10 = −0.28), and several other lipids, including LPE 22:5 (log10 = −0.13), LPE 20:4 (log10 = −0.11), and PS 18:0/18:1 (log10 = −0.09), were also downregulated. PE 18:0/20:4, PE 18:0/20:3, and SM 42:2 showed moderate decreases (log10 ranging from −0.05 to −0.06).

The treatment with ePS alone induced significant remodeling of the microglial lipidome in the absence of inflammatory stimulation (Figure 7c). The most prominent effect was a strong upregulation of two PS molecular species: PS 36:3 and PS 40:5 (log10(ePS/control) = 0.56 and 0.42). PI 18:0/16:1, whose level was also elevated in LPS-treated cells, showed a similar increase (log10 = 0.56). Several LPE, including LPE 20:1, LPE 22:2, and LPE 22:1, were also upregulated (log10 ranging from 0.45 to 0.21). Conversely, the ePS treatment led to a decrease in the level of a distinct set of PL. PI 18:1/20:4 level showed a significant reduction (log10 = −0.26), followed by LPE 22:5 (log10 = −0.13), LPE 20:4 (log10 = –0.10), and PS 18:0/18:1 (log10 = −0.10). Smaller decreases were observed for PE 18:1/20:4, PI 18:0/20:4; 18:1/20:3, and SM 42:2 (log10 ranging from −0.03 to −0.06).

## 3. Discussion

Microglia, as “brain-resident macrophages”, mediate both protective and deleterious responses to damage and pathogen-associated stimuli and are, therefore, critical regulators of the immune response in neurodegenerative diseases [15,16]. Microglia play a key role in activating neuroinflammation in response to inflammatory stimuli, releasing ROS upon harmful stimuli, which can lead to oxidative stress. The latter is a detrimental condition caused by an imbalance between the production of ROS and their physiological neutralization [16]. This phenomenon is a consequence of disrupted homeostasis, where the production of ROS becomes excessive or defense systems diminished [17]. When homeostasis is disrupted and microglia are excessively activated, ROS are accumulated, and neurons, being rich in lipids and having weak antioxidant activity, become easy targets. Therefore, search for new substances with antioxidant properties is currently one of the most promising areas of research. In this study, we assessed, for the first time, the effects of ether phosphatidylserines (ePS) from the soft coral *S. heterospiculatum* and total phospholipid extract (extPL) from this coral on the functional activity of HMC-3 cell line, paying special attention to their antioxidant potential, and the effect on the lipid composition of membranes.

When testing the cytotoxicity of ePS, we found that it exhibited no cytotoxic effect at any concentration but increased microglial cell proliferation at concentrations of 50 and 100 μg/mL. The increase in metabolic activity of microglial cells upon ePS treatment may be associated with their incorporation into membranes or with the activation of proliferative signaling pathways, which requires further study. Conversely, extPL at a concentration of 25 μg/mL increased cell proliferation but exhibited cytotoxic activity at concentrations of 50–100 μg/mL. It should be noted that the observed decrease in ROS and NO levels in microglial cells pre-treated with maximum concentrations of the extPL was likely associated with cell death, as demonstrated in the MTS analysis and manifested as specific changes in cell morphology.

LPS, also known as endotoxins, are structural components of the outer membrane of Gram-negative bacteria. LPS induces inflammation through activation of TLR4, which is expressed on immune cells such as macrophages on many other cell types [18]. LPS also affects the lipid homeostasis of cells and has been shown to shift the membrane FA composition toward SFA [19]. The authors demonstrated a synergistic effect of LPS and palmitic acid on HMC-3 cells, followed by increased levels of proinflammatory cytokines [19]. The observed increased level of saturated FA profile of LPS-stimulated HMC-3 cells (Table 1) is consistent with the known effects on microglial cell activation [19]. The accumulation of SFA, especially 16:0 and 18:0, can promote lipid raft formation and enhance TLR4 clustering, thereby potentiating proinflammatory signaling [18]. Pretreatment with ePS prevented the LPS-induced increase in SFA, restoring FA levels to a control-like state. Furthermore, the ePS and ePS + LPS groups had relatively higher PUFA contents than the control and LPS groups. These results suggest that ePS are able to maintain a normal microglial FA composition, thereby preventing excessive oxidative stress and inflammatory response.

We observed significant changes in PI molecular species composition in response to LPS stimulation. The PI molecular species (PI 18:0/20:4; 18:1/20:3 and PI 18:0/20:3) were likely subjected to active cleavage by phospholipase C, producing inositol phosphate and diacylglycerol, the secondary messengers that trigger proinflammatory reactions [20]. Concurrently, there was an increase in monounsaturated PI forms (PI 18:0/18:1, PI 18:0/16:1), representing a compensatory mechanism. A similar pattern was observed for LPE. Increased levels of LPE 20:1, LPE 18:1, and LPE 22:2 in LPS indicated excessive phospholipases A2 activity, which is implicated in inflammatory process [21]. The decrease in LPE 22:5 may be associated with its metabolism or oxidation. Treatment of cells with ePS before LPS exposure (ePS + LPS group) did not completely block but modulated the inflammatory process. Levels of precursors for proinflammatory PI (PI 18:0/20:4; 18:1/20:3, and PI 18:0/20:3) remained decreased, but the most notable effect was the reduction in PI 18:1/20:4 recorded from both the ePS and ePS + LPS groups (Figure 7b). The reduction in PI molecular species containing arachidonic acid (AA) (20:4n-6) was of particular importance, since this acid is a key precursor of proinflammatory eicosanoids such as prostaglandins, leukotrienes, and thromboxanes that enhance neuroinflammation [22]. Interestingly, our results are consistent with work [23]. They showed that LPS enhances AA release in macrophages by slowing down the transfer of AA into plasmalogens. In our study, we found that ePS treatment reduces the levels of AA-containing PI (PI 18:1/20:4 and PI 18:0/20:4). This suggests that ePS is able to influence the pool of signaling PI, which contributes to the observed reduction in ROS and NO production [24,25].

The antioxidant effect of ePS may be mediated by two mechanisms. First, ePS, containing an ether bond at the sn-1 position and PUFA at the sn-2 position, can act as free radical scavengers [26,27]. Second, as demonstrated by the lipid analysis, ePS modulate membrane composition, reducing the level of PI molecular species with PUFA in particular AA, which serve as precursors for the production of proinflammatory mediators. Interestingly, under the combined action of ePS and LPS (Figure 7b), we did not observe a complete restoration of these PI levels to those of LPS or control group, which may reflect the combined effects of LPS activation and ePS modulation. The presence of an ether bond at the *sn-1* position and very-long-chain PUFA (24:5) at the *sn-2* position in the isolated ePS is structurally unique and likely contributes to its antioxidant and modulated properties.

Structural identification of the ePS from *Sclerophytum heterospiculatum* was performed by HPLC-MS/MS, allowing determination of molecular mass, the presence of an ether bond in *sn-1* position, and FA composition (Figure S7). The identification of the 24:5 FA as tetracosapentaenoic acid with n-6 double bond is based on literature reports on the FA composition of soft corals [8,9]. Specifically, soft corals of the genera Sclerophytum (formerly Sinularia) have been shown to contain very-long-chain PUFA with n-6 and n-3 [8,9]. Nevertheless, it should be acknowledged that the double bound positions were not confirmed by independent chemical methods (NMR spectroscopy).

The current study had several limitations. First, our lipid analysis focused on the major phospholipid classes and did not include detailed profiling of sphingolipids such as ceramides. These molecules play a critical role in regulating apoptosis, proliferation, and inflammation in microglia [28]. Second, although we observed a significant increase in the molecular species PS 36:3 and PS 40:5 upon ePS treatment (Figure 7c), we cannot confidently conclude that this occurred due to the direct incorporation of exogenous *S. heterospiculatum* ePS into HMC-3 cell membranes. Also using the HMC3 cell line to model neuroinflammation, it is important to consider the serious limitations of this tool, which directly affect the interpretation of the results. Namely: 1. The cells express pericyte markers (PDGFRβ, NG2), and in the inactivated state, they weakly express MHCII, CD68, and CD11b markers. However, as stated on the manufacturer’s website, upon cell activation, the expression of microglial markers increases. 2. Reduced physiological activity: Compared to primary cells, HMC3 have low phagocytic activity and a distorted (“narrowed”) cytokine response. 3. Phenotypic drift: As with any immortal cell line, the properties of HMC3 change over time and vary from laboratory to laboratory, which reduces reproducibility.

In this study, we, for the first time, tested isolated ether phosphatidylserines (ePS) from the soft coral *S. heterospiculatum* for their biological activity in a HMC-3 model. Unlike the total phospholipid extract, ePS proved to be non-cytotoxic and suppressed the production of ROS, NO, and MDA in LPS-activated cells. The lipidomic analysis showed that ePS can modulate the lipid composition of microglial membranes, specifically increasing the content of endogenous PS with PUFA and reducing the level of PI 18:0/20:4, a precursor of proinflammatory mediators. These findings indicate ether phospholipids from marine organisms as promising for the development of new neuroprotective agents to mitigate oxidative stress and neuroinflammation.

## 4. Materials and Methods

### 4.1. Extraction and Isolation of Ether Phosphatidylserine

Chloroform, methanol, acetone, ethanol, benzene and 28% NH_4_OH for lipid extraction and thin-layer chromatography (TLC) were of analytical grade.

The colonies of soft coral *Sclerophytum heterospiculatum* were maintained in a 500 L tank with aerated seawater collected from Peter the Great Bay, Russia (43°19.8′ N, 131°91.8′ E). The colonies were illuminated a metal halide lamp fitted with a 250 W Aqualine, providing a photosynthetic photon flux density of 200 μmol/m^2^/s (Aqua Medic, Bissendorf, Germany). The photoperiod was set at 12 h light/12 h dark. To obtain a total lipid extract, one large colony weighing 20 g was taken. All experiments and their repetitions were conducted with the same batch of isolated ePS and extPL.

The total lipid extract was prepared according to the method [29] with minor modifications. Total lipids were stored at −20 °C. Phospholipid fractions were obtained according to the method [30] with minor modifications. The total lipid extract (0.15 g) was loaded onto the silica gel (Sigma Aldrich, Saint Louis, MO, USA) column and then washed with the following solvents: 10 volumes of chloroform (175 mL), 40 volumes of acetone (700 mL), and 10 volumes of methanol:water (9:1) (200 mL). The latter fraction was eluted into 20 fractions of 10 mL each. For the detection of ether phosphatidylserine (ePS), all 20 fractions were analyzed by TLC. The plate was developed along its entire length with a mixture of chlorform:methanol:28% NH_4_OH:benzene (65:30:6:10, *v*/*v*). After drying in an air stream, the plate was then sprayed with 10% sulfuric acid/methanol and heated at 240 °C for 10 min (Figures S8 and S9). Next, we scraped the ePS zone (only fraction 10) off the plate, redissolved it in chloroform, and evaporated it on a rotary evaporator. The isolated ePS fraction was also analyzed by HPLC-MS/MS on a Shimadzu LCMS-IT-TOF mass spectrometer (Shimadzu, Kyoto, Japan). The isolated ePS fraction was dissolved in a small volume of chloroform, and stored at −40 °C in a glass vial with a Teflon cap. Working solutions in ethanol were prepared immediately before use.

### 4.2. Cell Lines and Culture Conditions

The HMC-3 cell line (CRL-3304, ATCC, UK) was used as a model object. Cells were cultured under standard conditions in DMEM/F12 (Thermo Fisher Scientific, Waltham, MA, USA) supplemented with 10% FBS (Thermo Fisher Scientific, USA), essential amino acids (Gibco, Carlsbad, CA, USA), and 5% penicillin/streptomycin (Thermo Fisher Scientific, USA). The cells were incubated at 37 °C in a humidified environment with 5% CO_2_ (MCO-18AIC, Sanyo, Osaka, Japan). HMC-3 cells were pre-seeded in a 96-well plate (1 × 10^5^ cells/cm^2^) to investigate the cytotoxicity (MTS) and antioxidant activity (NO and ROS), and in a T-25 culture dish to analyze membrane lipids and MDA production. Also, to study changes in cell morphology under exposure to the compounds under study, microphotographs of control and treated cells were taken under a Zeiss AxioVert A1 microscope equipped with a CCD camera (AxioCam HRc) (Carl Zeiss, Oberkochen, Germany). All experiments on microglial cell culture were repeated three times independently.

### 4.3. MTS Assay

To analyze the cytotoxic effects of ePS and extPL, MTS staining was used (Abcam, ab197010, Waltham, MA, USA) [31]. Cells were plated in 96-well plates and incubated for 1 h in standard culture medium. The standard medium was then replaced with a medium supplemented with the test compounds and incubated for 72 h. Cells in standard medium without the supplemented compounds served as a control. The final concentration of the solvent (ethanol) at the maximum concentration did not exceed the permissible 0.5%. Absorbance at 490 nm was measured using a Bio-Rad iMark microplate reader (Bio-Rad, Hercules, CA, USA). Cell viability was calculated relative to the untreated control cells and presented as a percentage of control.

### 4.4. NO and ROS Measurement

The antioxidant activity of ePS and extPL was examined in a culture of HMC-3 cells activated by bacterial lipopolysaccharide (LPS, *E. coli* O111:B4, L4130, Sigma Aldrich, USA). One hour after cell adhesion, the culture medium was enriched with the compounds at different concentrations. The cells were then stimulated by adding LPS (1 µg/mL). The positive control consisted of cells activated only with LPS, and the negative control consisted of cells in the standard medium. After 24 h of incubation, 20 µL of 2,7-dichlorodihydrofluorescein diacetate solution (D399, Invitrogen, Carlsbad, CA, USA) was added in accordance with the manufacturer’s instructions to assess ROS activity [32], and 10 μM DAF-FM diacetate (Thermo Fisher Scientific, USA) was added to assess NO activity [33]. Fluorescence intensity was measured using a SPARK TECAN 10 M plate reader (TECAN, Männedorf, Switzerland) for ROS at λex/λem = 485/518 nm and for NO at λex/λem = 460/524 nm. Results are presented as a percentage of negative control.

### 4.5. MDA

A Lipid Peroxidation Assay Kit (MAK085, Sigma, St. Louis, MO, USA) was used to measure the levels of malondialdehyde (MDA) in LPS-activated HMC-3 cell culture. After the cells adhered to the culture dish, the standard medium was replaced with a medium enriched in studied compounds. The cells were then activated by adding LPS (1 µg/mL). The cells in the standard culture medium served as negative control, whereas the cells activated only with LPS served as positive control. Cells were passaged after 24 h to a Versene/EDTA solution (HIMedia, Thane, Maharashtra, India), then centrifuged at 400× *g* for 8 min, followed by removal of the supernatant. The cell pellet itself was immediately frozen in liquid nitrogen and stored at −80 °C until further use. After defrosting, the cell pellet was homogenized with ultrasound, and all subsequent procedures were carried out according to the manufacturer’s recommendations. Optical density was measured using a microplate reader (Bio-Rad, USA) at a wavelength of 532 nm. Results are presented relative to negative control without LPS added.

### 4.6. GC-FID and HPLC–MS/MS Analysis of Lipids of Microglial Cells

For HPLC, LC-MS grade hexane, 2-propanol, triethylamine, and formic acid were used.

Fatty acid methyl esters (FAME) from HMC-3 culture cell lipids were prepared using the method of Carreau and Dubak [34]. FAMEs were analyzed by gas chromatography on a GC-2010 chromatograph (Shimadzu, Japan) with a flame ionization detector and a Supelcowax 10 capillary column with a size of 30 m × 0.25 mm (internal diameter) (Supelco, Bellefonte, PA, USA) according to the method [35]. The analysis was carried out under the following conditions: column temperature, 205 °C; injector and detector temperature, 240 °C. Helium was used as the carrier gas. FAME peaks were identified by comparing the retention times of individual fatty acid esters and equivalent chain lengths with the respective standards (a mixture of PUFA from menhaden oil, Supelco, USA). FAME peaks were identified by the retention times of individual fatty acid esters by comparing their carbon equivalent length numbers with authentic standards [36].

Membrane lipid analysis was performed in microglial cells pre-incubated in a medium enriched with the studied compounds for 72 h. After incubation, the cells were dissociated using a Versene/EDTA solution (HIMedia, Maharashtra, India), frozen in liquid nitrogen, and stored at −80 °C until further use. The content and structures of PL molecular species were analyzed on a HPLC system coupled to a high-resolution tandem mass spectrometer according to the method [9]. Total lipids were separated on a Shim-Pack diol column (2.1 mm × 150 mm, particle size 3 μm) (Shimadzu, Japan) using a Nexera-e chromatography system (Shimadzu, Japan). Solvent system A (2-propanol:hexane:H_2_O:formic acid:NH_4_OH (28%):triethylamine, 28:72:1.5:0.1:0.05:0.02, *v*/*v*) and solvent system B (2-propanol:H_2_O:formic acid:NH_4_OH (28%):triethylamine, 100:1.5:0.1:0.05:0.02, *v*/*v*) were used as eluents. The percentage of system B in the total solvent flow was programmed as follows: 0 to 20% (7 min), 20 to 100% (5 min), 100% (5 min), 100 to 0% (0.1 min), and 0% (10 min). The flow rate was 0.2 mL/min. Lipids were detected on a high-resolution tandem mass spectrometer LCMS-IT-TOF (Shimadzu, Japan). Analysis was performed in the electrospray ionization (ESI) mode with simultaneous detection of signals of positive and negative ions. Scanning was performed in an *m*/*z* range of 100–1200. The source voltage was −3.5 kV for negative ions and 4.5 kV for positive ions. The temperature of the ion source was 250 °C, the dry gas (N_2_) pressure, 200 kPa; and the flow rate of nebulizing gas (N_2_), 1.5 L/min. Argon (0.003 Pa) was used in the collision chamber of the mass spectrometer. Percentages of certain molecular species of each lipid class were calculated on the basis of the peak area of negative ions [M–H]^–^, except PC that was determined using the peak area of positive ions [M+H]^+^. We used the following lipid standards: PC (12:0/12:0), PE (16:0/16:0), PS (16:0/18:0), PG (16:0/16:0), and LPE (18:0) (Avanti Polar Lipids, Alabaster, AL, USA). The molecular species of PL were identified manually and also according to these methods [37,38]. The identification of the major molecular species is presented in the Supplementary Materials (Figures S1–S6).

### 4.7. Statistical Analysis

Values of lipid contents are presented as mean ± SD (standard deviation) for three biological samples. Raw data were used after being tested for normality of distribution (Shapiro–Wilk test). Differences in mean concentrations of PL molecular species (% of lipid extract) between the groups “Control”, “LPS”, “ePS”, and “ePS + LPS” were analyzed by one-way ANOVA by post hoc Tuckey’s HSD test. A probability level of *p* < 0.05 was considered statistically significant. For in vitro studies, values are presented as mean  ±  SEM (standard error of the mean) for four biological samples. The Shapiro–Wilk test confirmed normal data distribution. Differences between groups for the in vitro study were analyzed by Student’s *t*-test. Differences were considered statistically significant at *p*  <  0.05. All statistical calculations were performed and graphs composed using the GraphPad Prism 4.00 software (GraphPad Software, San Diego, CA, USA) and the R statistical program version 4.4.1.

## Figures and Tables

**Table 1:** Fatty acid composition (% of total fatty acids) of total lipids from HMC-3 human microglial cells under different experimental conditions. Cells were treated for 72 h under the following conditions: Control, LPS, ePS, ePS + LPS. Fatty acid methyl esters (FAMEs) were analyzed by GC-FID. Data are presented as mean ± SD; *n* = 3 (number of samples analyzed); * *p* ˂ 0.05 (one-way ANOVAby post hoc Tuckey’s HSD test). Abbreviations: Control—untreated control cells; LPS—cells treated with lipopolysaccharide (1 µg/mL); ePS—cells treated with only ePS (25 µg/mL); ePS + LPS—ePS pretreatment (25 µg/mL) + LPS (1 µg/mL); SFA—saturated fatty acids; MUFA—monounsaturated fatty acids; PUFA—polyunsaturated fatty acids.

	Control	LPS	ePS	ePS + LPS	ANOVA *p* Value	ANOVA F
14:0	6.771 ± 3.477	6.87 ± 0.128	3.924 ± 0.835	3.999 ± 0.399	0.131	2.524
15:0	3.004 ± 0.98	2.264 ± 0.16	1.777 ± 0.71	1.203 ± 0.08 *	0.035	4.711
16:0	31.954 ± 5.2	46.323 ± 6.76 *	33.202 ± 5.11	40.365 ± 4.32	0.038	4.578
16:1 (n-9)	5.384 ± 2.05	2.862 ± 3.5	4.974 ± 1.66	1.911 ± 1.65	0.282	1.521
16:1 (n-7)	1.438 ± 0.26	0.792 ± 0.74	1.015 ± 0.91	0.91 ± 0.32	0.626	0.613
17:0	1.316 ± 0.85	1.365 ± 0.31	0.669 ± 0.14	1.115 ± 0.2	0.321	1.364
18:0	17.497 ± 1.94	24.382 ± 2.93 *	18.025 ± 3.65	23.097 ± 2.18 *	0.033	4.826
18:1 (n-9)	10.654 ± 6.46	3.431 ± 4.46	13.853 ± 4.04	6.578 ± 6.36	0.176	2.117
18:1 (n-7)	4.018 ± 2.61	1.026 ± 1.15	5.552 ± 1.79	2.788 ± 2.53	0.135	2.482
18:2 (n-6)	1.556 ± 1.32	0.693 ± 0.9	1.056 ± 0.49	0.524 ± 0.39	0.504	0.851
18:4 (n-3)	0.557 ± 0.25	0.417 ± 0.18	0.319 ± 0.07	0.564 ± 0.13	0.311	1.404
20:0	0.746 ± 0.21	0.694 ± 0.2	0.419 ± 0.09	0.64 ± 0.08	0.130	2.542
20:3 (n-6)	1.985 ± 1.09	0.431 ± 0.18	0.525 ± 0.4	1.029 ± 0.18 *	0.052	4.001
20:4 (n-6)	0.564 ± 0.15	0.733 ± 0.71	1.521 ± 0.32 *	0.458 ± 0.25	0.044	4.314
20:4 (n-3)	0.688 ± 0.33	0.665 ± 0.35	0.429 ± 0.12	0.618 ± 0.22	0.648	0.575
22:1 (n-9)	1.78 ± 0.83	1.304 ± 0.2	1.559 ± 0.79	1.69 ± 0.7	0.839	0.279
22:5 (n-3)	1.829 ± 1.19	0.232 ± 0.06	0.878 ± 0.43	1.056 ± 0.74	0.143	2.407
22:6 (n-3)	0 ± 0	1.331 ± 0.59 *	1.582 ± 1.29	1.546 ± 0.97	0.047	4.288
Other	11.295 ± 3.59	4.183 ± 2.5	8.161 ± 2.28	9.385 ± 3.29	0.090	3.083
SFA	61.288 ± 8.11	81.899 ± 9.95 *	58.015 ± 8.41	70.42 ± 6.87	0.033	4.866
MUFA	23.274 ± 9.86	9.415 ± 9.66	26.954 ± 6.53	13.878 ± 10.13	0.148	2.360
PUFA	8.958 ± 1.9	5.806 ± 1.25	7.868 ± 2.65	7.486 ± 2.07	0.357	1.242

## Data Availability

The data presented in this study are available on request from the corresponding author.

## Supplementary Materials

The following supporting information can be downloaded at: https://www.mdpi.com/article/10.3390/md24060188/s1, Table S1: MTS assay of ePS and extPL on human microglia cell (HMC-3); Table S2: Antioxidant activity (ROS, NO and MDA) of ePS and extPL toward human microglial cell (HMC-3); Table S3: The content of molecular species of phospholipids of human microglia HMC-3 under exposure to LPS, ePS and ePS + LPS; Figure S1: Electrospray ionization mass spectra of proposed 18:1/18:1 PE; Figure S2: Electrospray ionization mass spectra of proposed 16:0/18:1 PC; Figure S3: Electrospray ionization mass spectra of proposed 18:0/18:1 PS; Figure S4: Electrospray ionization mass spectra of proposed 18:0/20:3 PI; Figure S5: Electrospray ionization mass spectra of proposed 20:4 LPE; Figure S6: Electrospray ionization mass spectra of proposed 18:1/18:1 PG; Figure S7; Electrospray ionization mass spectra of proposed 18:0e/24:5 PS; Figure S8: Thin-layer chromatography of the isolated 1–12 fractions and the standard PS 18:0/18:1; Figure S9: Thin-layer chromatography of the isolated 13–20 fractions and the standard PS 18:0/18:1.

## Abbreviations

The following abbreviations are used in this manuscript:

AA	Arachidonic acid
CNS	Central nervous system
FA	Fatty acid
GC-FID	Gas–liquid chromatography with flame ionization detection
HMC-3	Human microglial clone 3 cells
HPLC-MS/MS	High-performance liquid chromatography with tandem mass spectrometry
LPE	Lysephosphatidylethanolamine
LPS	Lipopolysaccharide
MDA	Malondialdehyde
MUFA	Monounsaturated fatty acid
NO	Nitric oxide
PC	Phosphatidylcholine
PE	Phosphatidylethanolamine
PG	Phosphatidylglycerol
PI	Phosphatidylinositol
PL	Phospholipids
PS	Phosphatidylserine
PUFA	Polyunsaturated fatty acid
ROS	Reactive oxygen species
SFA	Saturated fatty acid
SM	Sphingomyelin
TPA	Tetracosapolyenoic fatty acid
TLC	Thin-layer chromatography
