# Supplementary material for: Ether Phosphatidylserine from Soft Coral Sclerophytum heterospiculatum Reveals Antioxidant Activity and Modulates Lipid Composition in LPS-Activated Human Microglial HMC-3 Cells
Source: Mar Drugs. 2026 May 23;24(6):188. doi: 10.3390/md24060188 (PMC13302565; doi:10.3390/md24060188)
Supplement: Supplementary file 1 [file marinedrugs-24-00188-s001.zip › marinedrugs-4268086-supplementary.pdf]

Ether Phosphatidylserine from Soft Coral *Sclerophytum heterospiculatum* Reveals Antioxidant Activity and Modulates Lipid Composition in LPS-Activated Human Microglial HMC-3 Cells

Elena T. Bizikashvili *, Arina I. Ponomarenko, Ekaterina V. Ermolenko and Igor V. Manzhulo

A.V. Zhirmunsky National Scientific Center of Marine Biology, Far Eastern Branch, Russian Academy of Sciences, ul. Palchevskogo 17, 690041 Vladivostok, Russia; arina.ponomarenko.93@mail.ru (A.I.P.); ecrire_711@mail.ru (E.V.E.); i-manzhulo@bk.ru (I.V.M.)

*Correspondence: bilielena801@gmail.com; Tel.: +7-423-2310905

Table S1. MTS assay of ePS and extPL on human microglia cell (HMC-3)

	MTS	
	extPL	ePS
Control	102.787±2.045	97.213±0.39
0.39 μg/ml	95.738±1.781	99.59±0.246
0.78 μg/ml	93.525±1.438	100.82±2.305
1.56 μg/ml	94.18±1.832	98.361±1.011
3.12 μg/ml	99.098±2.897	102.705±1.04
6.25 μg/ml	102.869±1.177	105.656±1.552
12.5 μg/ml	107.377±3.637	107.131±2.48
25 μg/ml	117.869±3.677*	103.689±1.45
50 μg/ml	79.344±2.056*	110.82±2.379*
100 μg/ml	39.426±1.904*	113.443±3.405*

Data were presented as mean ± SEM, $n = 18$; * $p < 0.05$ vs. Control group

Table S2. Antioxidant activity (ROS, NO and MDA) of ePS and extPL toward human microglial cell (HMC-3)

	NO		ROS		MDA	
	extPL	ePS	extPL	ePS	extPL	ePS
Control	99.999±1.292	99.999±1.292	95.332±8.07	104.677±9.831	97.817±4.074	97.817±4.074
LPS	138.087±5.8*	133.229±7.214*	220.509±15.696*	263.957±26.981*	119.358±2.358*	119.358±2.358*
0.39 μg/ml	196.894±9.549*	189.546±12.944*	245.333±18.616*	269.918±11.484*	—	—
0.78 μg/ml	182.412±8.798*	194.549±6.12*	216.7±12.077*	222.267±7.512*	—	—
1.56 μg/ml	169.317±3.912*	162.55±7.087*	236.572±22.721*	253.557±4.859*	—	—
3.12 μg/ml	152.082±6.227*	153.958±9.395*	244.353±18.262*	238.618±15.351*	—	—
6.25 μg/ml	143.487±3.777*	155.028±11.411*	249.221±8.075*	226.168±5.338*	—	—
12.5 μg/ml	124.042±4.636*	138.604±10.598*	190.004±6.97*	233.125±5.246*	—	—
25 μg/ml	115.886±3.224*+	130.31±3.365*	167.837±9.454*+	200.57±14.651*	58.91±0.338*+	76.275±2.214*+
50 μg/ml	108.112±2.155*+	115.784±3.593*+	122.619±2.669*+	151.048±13.535*+	—	—
100 μg/ml	112.984±2.889*+	109.207±1.752*+	125.515±8.849*+	127.698±9.638*+	—	—

Data were presented as mean ± SEM, $n = 18$; * $p < 0.05$ vs. Control group; + $p < 0.05$ vs. LPS group

Table S3. The content of molecular species of phospholipids of human microglia HMC-3 under exposure to LPS, ePS and ePS+LPS

	Molecular species of PL	Control	LPS	ePS	ePS+LPS	ANOVA p value	ANOVA F
Phosphatidylethanolamines (% from total PE)							
1	16:1/16:0 PE	2.296±0.162	2.738±0.328	2.841±0.19*	2.956±0.553	0,018	6,17
2	16:1/18:1 PE	4.248±0.207	4.209±0.501	4.336±0.133	4.314±0.651	0,981	0,056
3	16:0/18:1 PE	9.739±1.099	9.452±0.843	8.752±0.442	9.18±0.823	0,548	0,758
4	16:0/20:4 PE	2.107±0.044	2.155±0.17	2.38±0.077*	2.346±0.285	0,034	4,876
5	18:1/18:1 PE	20.683±1.719	19.965±0.295	20.468±0.926	18.027±1.15	0,075	3,366
6	18:1/18:0 PE	16.372±1.097	17.633±1.919	18.202±0.556	18.129±0.74	0,287	1,498
7	18:1/20:5 PE	2.102±0.058	2.16±0.555	2.165±0.093	3.148±1.653	0,442	0,998
8	18:1/20:4 PE	6.175±0.133	5.495±0.198*	5.769±0.107*	5.709±0.502	0,007	8,717
9	18:0/20:4 PE	10.73±0.387	9.868±0.44	10.062±0.177	9.555±0.468*	0,031	4,991
10	18:0/20:3 PE	8.271±0.466	7.19±0.524	8.423±0.223	7.203±0.244*	0,006	8,896
11	18:1/20:1; 18:0/20:2 PE	2.801±0.281	3.843±0.269*	2.256±0.93	2.277±1.123	0,033	4,819
12	16:0/22:1 PE	0.502±0.411	0.258±0.225	0.513±0.118	0.746±0.37	0,344	1,284
13	18:1/22:6 PE	2.474±0.376	2.05±1.115	2.545±0.18	3.917±1.947	0,284	1,514
14	18:0/22:6 PE	5.061±0.136	5.393±0.246	4.912±0.242	5.827±1.66	0,587	0,683
15	18:0/22:5 PE	3.752±0.281	4.269±0.175	3.372±0.259	3.716±0.108	0,093	3,028
16	18:1/22:3; 18:0/22:4 PE	1.64±0.398	1.924±0.199	1.871±0.217	1.548±0.514	0,543	0,769
17	18:0/22:3 PE	1.048±0.323	1.397±0.273	1.132±0.038	1.403±0.276	0,275	1,553
Phosphatidylcholines (% from total PC)							
1	14:0/16:0 PC	4.409±0.803	5.749±0.288	4.884±0.298	5.006±0.482	0,068	3,525
2	16:0/16:1 PC	14.307±0.331	13.474±0.362*	14.968±0.198*	14.543±0.379	0,003	11,200
3	16:0/16:0 PC	4.699±1.14	5.215±0.257	3.982±0.764	3.732±1.232	0,267	1,586
4	34:1e PC	1.586±0.523	2.162±0.118	1.8±0.142	1.86±0.387	0,291	1,484
5	33:1 PC	1.321±0.044	1.493±0.244	1.543±0.177	1.471±0.13	0,443	0,996
6	16:1/18:1 PC	10.245±0.086	9.484±0.56	10.25±0.206	10.363±0.211	0,101	2,912
7	16:0/18:1 PC	31.745±1.526	29.878±0.789	29.741±1.75	30.624±1.27	0,332	1,326
8	18:1/18:1 PC	21.608±0.384	21.064±0.205	21.992±0.156	21.246±0.338	0,178	2,103
9	18:1/18:0 PC	10.079±0.659	11.479±0.455	10.84±0.521	11.155±1.317	0,258	1,628
Phosphatidylglycerols (% from total PG)							
1	16:1/18:1 PG	3.76±1.594	3.918±0.723	5.562±0.38	4.009±1.489	0,273	1,558
2	16:0/18:1 PG	2.897±1.223	4.262±0.864	4.866±0.712	4.595±1.651	0,249	1,674
3	18:1/18:2 PG	9.524±0.486	9.351±0.218	9.784±0.553	8.75±1.243	0,409	1,086
4	18:1/18:1 PG	44.702±0.928	46.897±2.829	44.399±1.392	41.129±3.855	0,121	2,644
5	18:2/20:2 PG	7.956±0.572	7.331±0.446	7.04±0.29	7.235±0.65	0,222	1,818
6	18:1/20:2 PG	15.651±0.987	15.398±0.228	14.788±0.687	14.492±1.045	0,332	1,325
7	18:1/22:6 PG	8.625±0.922	7.466±0.383	7.623±0.078	13.474±7.925	0,285	1,508

8	18:1/22:5 PG	6.885±0.612	5.377±0.698*	5.938±0.836	6.317±0.628	0,042	4,472
Phosphatidylserines (% from total PS)							
1	18:0/16:1 PS	6.238±4.641	9.199±1.847	9.076±1.611	9.315±4.27	0,647	0,576
2	36:3 PS	2.015±2.348	6.998±3.55	7.31±0.53*	6.997±2.087	0,043	4,424
3	18:0/18:1 PS	75.926±3.41	71±2.9	60.336±1.846*	61.416±5.799*	0,002	11,995
4	40:5 PS	4.378±0.176	5.425±0.959	11.451±3.134*	10.156±4.183	0,029	5,124
5	18:0/22:6 PS	11.444±1.502	7.378±2.426	11.825±1.008	12.116±4.266	0,169	2,173
Phosphatidylinositols (% from total PI)							
1	18:0/16:1 PI	1.401±0.535	5.418±1.546*	5.048±0.783*	5.76±1.349*	0,004	9,630
2	18:1/20:4 PI	18.302±4.043	14.139±3.404	10.038±1.41*	9.703±1.284*	0,017	6,214
3	18:0/20:4; 18:1/20:3 PI	35.706±1.388	28.28±2.288*	31.688±1.117*	29.369±2.843*	0,009	7,860
4	18:0/20:3 PI	25.896±3.024	19.795±1.173*	27.293±2.377	23.516±3.825	0,046	4,193
5	18:0/18:1 PI	2.532±2.086	10.185±0.788*	6.076±1.609	9.483±3.644*	0,011	7,123
6	18:1/18:1 PI	16.164±2.565	22.182±1.408*	19.857±1.128	22.169±2.585*	0,020	5,854
Lysophosphatidylethanolamines (% from total LPE)							
1	16:0 LPE	3.371±0.703	3.693±0.816	4.877±1.053	3.046±0.938	0,140	2,428
2	18:2 LPE	0.297±0.013	0.369±0.429	0.7±0.607	0.892±0.622	0,441	1,001
3	20:4 LPE	27.526±1.848	24.507±1.035	22.049±0.461*	21.443±2.058*	0,004	10,289
4	20:1 LPE	1.735±0.084	4.626±1.393*	4.905±0.918*	4.841±1.359*	0,018	6,077
5	22:6 LPE	15.633±2.671	13.537±2.583	12.615±0.376	20.064±13.152	0,574	0,707
6	22:5 LPE	16.473±0.69	13.323±0.712*	12.118±0.489*	12.145±1.413*	0,001	15,668
7	22:4 LPE	3.786±0.706	6.298±0.439*	4.465±1.088	3.74±0.96	0,018	6,151
8	18:1 LPE	11.923±0.154	14.401±0.875*	14.003±1.68	13.568±1.974	0,0133	6,893
9	20:3 LPE	12.106±1.489	9.884±3.439	14.585±1.532	11.557±1.933	0,159	2,259
10	22:3 LPE	4.239±1.699	5.047±1.21	4.366±0.507	4.326±1.619	0,873	0,230
11	22:2 LPE	1.099±0.421	2.118±0.457*	2.393±0.202*	1.912±0.774	0,050	4,029
12	22:1 LPE	1.81±0.566	2.198±1.059	2.924±0.191*	2.465±0.429	0,0383	4,581
Sphingomyelines (% from total SM)							
1	40:2 SM	10.777±5.323	16.359±1.766	17.04±0.543	16.029±2.323	0,118	2,681
2	42:3 SM	69.397±4.651	61.625±2.068	59.794±1.253*	60.573±1.561*	0,009	7,872
3	42:2 SM	19.827±8.598	22.016±2.409	23.166±0.731	23.399±1.403	0,764	0,389

Data were presented as mean ± SD, $n = 3$; * $p < 0.05$ vs. Control group

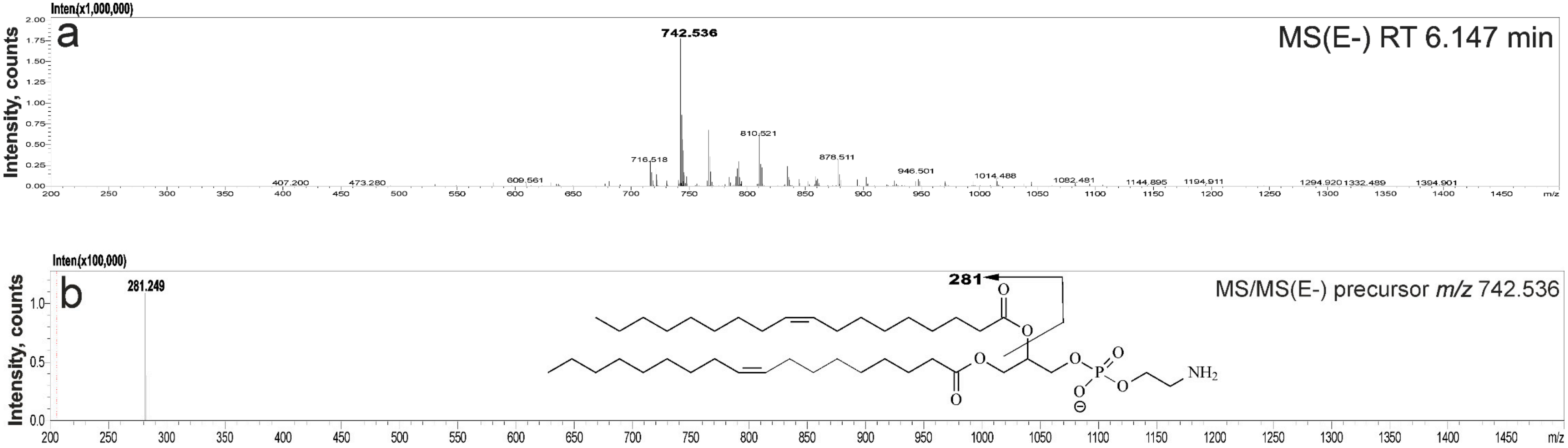

Fig. S1 Electrospray ionization mass spectra of proposed 18:1/18:1 PE. The total lipids of the HMC-3 were analyzed by high-performance liquid chromatography with tandem mass spectrometry (HPLC-MS/MS) in negative ion mode. **(a)** Mass spectrum for compounds eluting between 5.5-6.8 min. At tR = 6.147 min, the negative ion $[M–H]^{-}$ at *m/z* 742.536 was annotated as $[C_{41}H_{78}NO_8P]^{-}$ (calculated 742.539). **(b)** MS/MS spectrum of the precursor ion at *m/z* 742.536. Diagnostic fragments include the carboxylate anion of 18:1 (*m/z* 281.249). The predicted structure of 18:1/18:1 PE.

Based on the MS/MS fragmentation scheme, this molecular species was identified as diacyl-glycerophosphoethanolamine 18:1/18:1 PE.

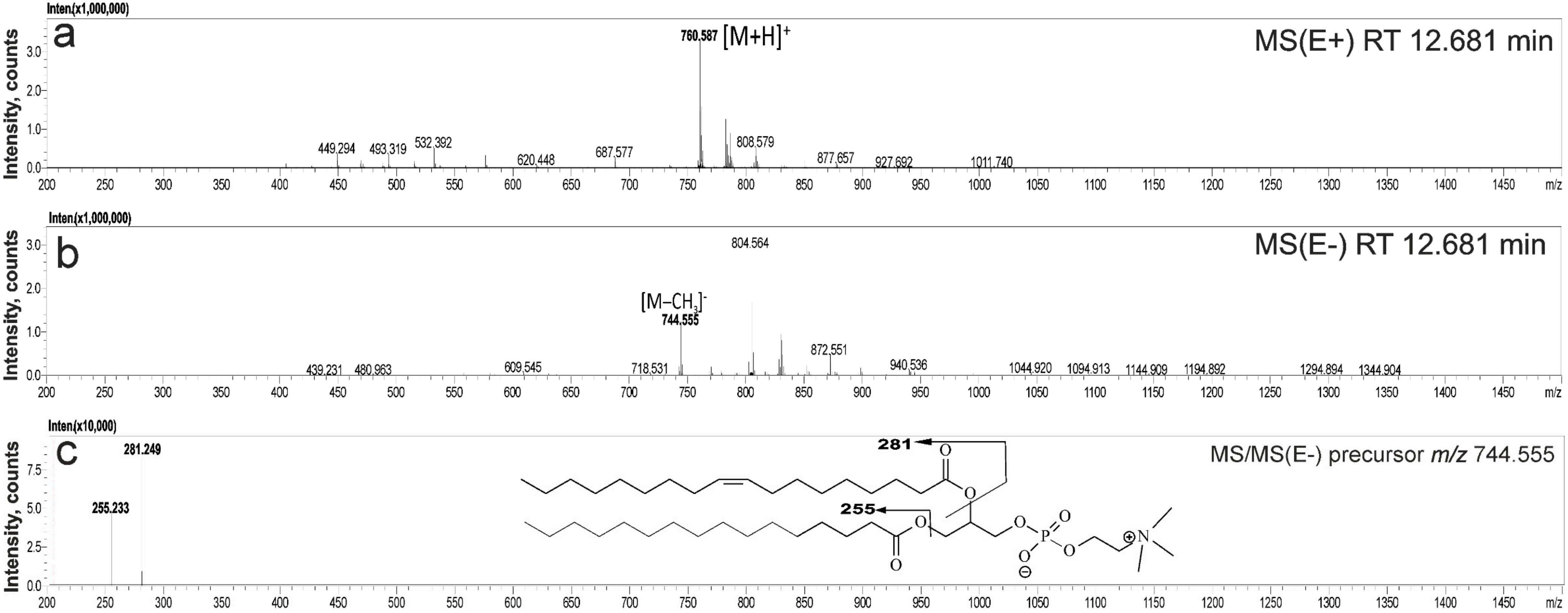

Fig. S2 Electrospray ionization mass spectra of proposed 16:0/18:1 PC. The total lipids of the HMC-3 were analyzed by high-performance liquid chromatography with tandem mass spectrometry (HPLC-MS/MS) in negative and positive ion modes. **(a)** Mass spectrum in positive ion mode for compounds eluting between 11.9-16.5 min. At tR = 12.681 min, the positive ion $[M+H]^+$ at *m/z* 760.587 was annotated as $[C_{42}H_{82}NO_8P]^+$ (calculated 760.585). **(b)** Mass spectrum in negative ion mode showing the methyl adduct $[M\text{-}CH_3]^-$ at *m/z* 744.555 (calculated 744.554). **(c)** MS/MS spectrum of the precursor ion at *m/z* 744.607. The carboxylate anion of 18:1 at *m/z* 281.249 and anion of 16:0 at *m/z* 255.233 confirms the acyl chain composition. The predicted structure of 16:0/18:1 PC.

Based on the fragmentation scheme, this molecular species was identified as diacyl-glycerophosphocholine 16:0/18:1 PC.

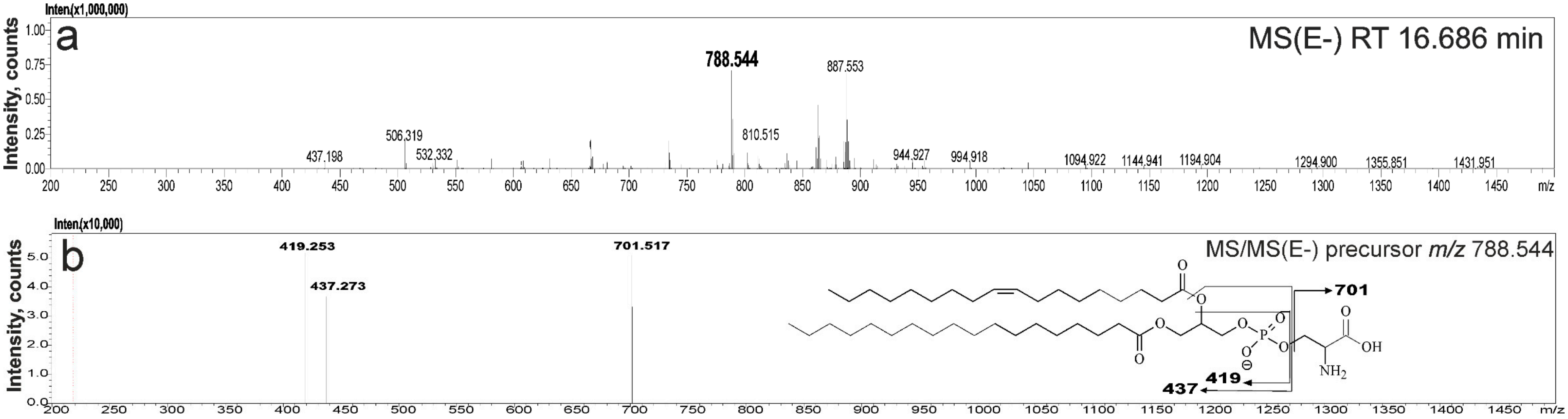

Fig. S3 Electrospray ionization mass spectra of proposed 18:0/18:1 PS. The total lipids of the HMC-3 were analyzed by high-performance liquid chromatography with tandem mass spectrometry (HPLC-MS/MS) in negative ion modes. **(a)** Mass spectra for compounds eluting between 16.6-17.7 min. At tR = 16.686 min, the negative ion $[M–H]^-$ at *m/z* 788.544 was annotated as $[C_{42}H_{80}NO_{10}P]^-$ (calculated 788.544). **(b)** MS/MS spectrum of the precursor ion at *m/z* 788.544. Diagnostic fragments include the loss of 87 (serine head group) at *m/z* 701. 517 and fragments at *m/z* 419.253 and *m/z* 437.273, confirming the 18:0 acyl chain. The predicted structures of 18:0/18:1.

Based on the MS/MS fragmentation scheme, this molecular species was identified as diacyl-glycerophosphoserine 18:0/18:1 PS.

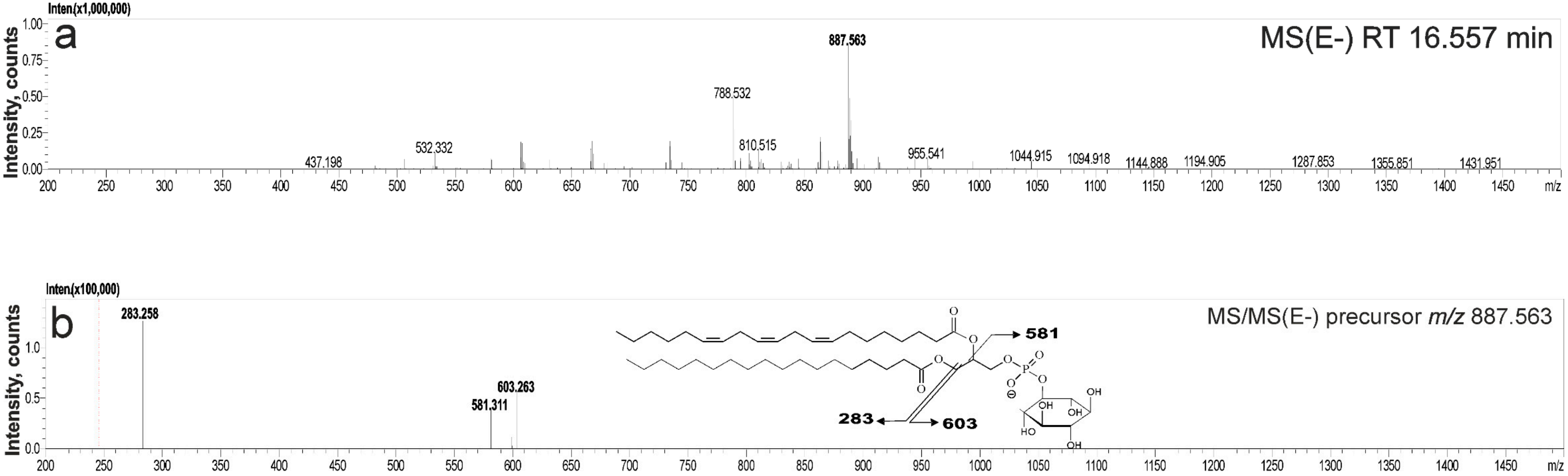

Fig. S4 Electrospray ionization mass spectra of proposed 18:0/20:3 PI. The total lipids of the HMC-3 were analyzed by high-performance liquid chromatography with tandem mass spectrometry (HPLC-MS/MS) in negative ion modes. **(a)** Mass spectrum for compounds eluting between 16.5-17.9 min. At tR = 16.557 min, the negative ion $[M–H]^–$ at *m/z* 887.563 was annotated as $[C_{47}H_{85}O_{13}P]^–$ (calculated 887.565). **(b)** MS/MS spectrum of the precursor ion at at *m/z* 887.553. The carboxylate anion of 18:0 at *m/z* 283.258 and anions without acyl fragments 20:3 at *m/z* 581.311 and anions without acyl fragments 18:0 at *m/z* 603.263. The predicted structure of 18:0/20:3.

Based on the MS/MS fragmentation scheme, this molecular species was identified as diacyl-glycerophosphoinositol 18:0/20:3 PI.

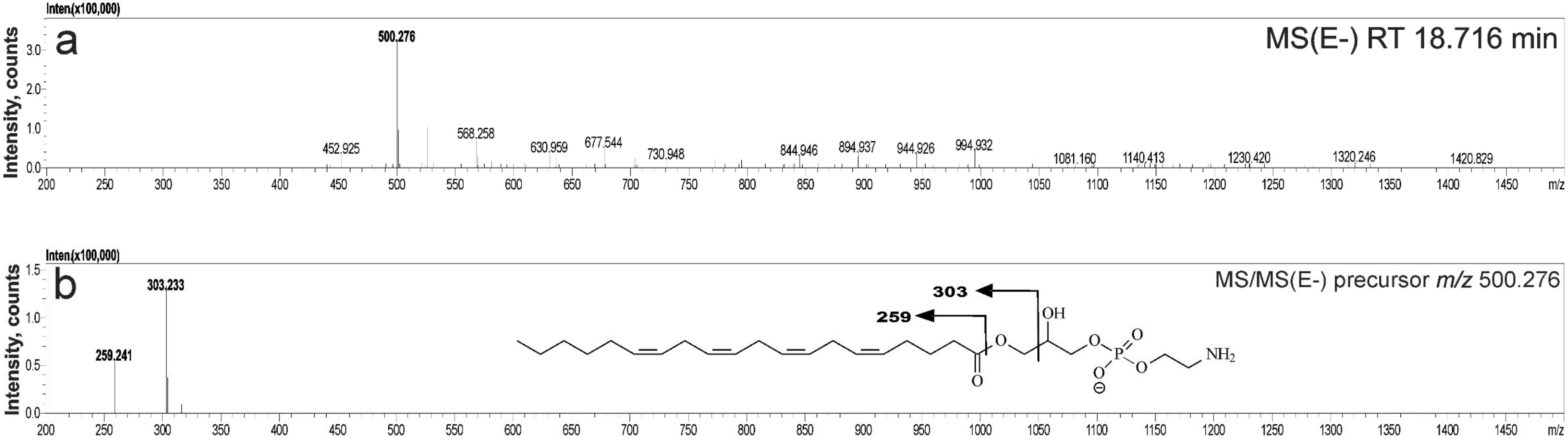

Fig. S5 Electrospray ionization mass spectra of proposed 20:4 LPE. The total lipids of the HMC-3 were analyzed by high-performance liquid chromatography with tandem mass spectrometry (HPLC-MS/MS) in negative ion modes. **(a)** Mass spectrum for compounds eluting between 17.9-19.1 min. At tR = 18.716 min, the negative ion $[M–H]^-$ at *m/z* 500.276 was annotated as $[C_{25}H_{44}NO_4P]^-$ (calculated 500.278). **(b)** MS/MS spectrum of the precursor ion at at *m/z* 500.276. The carboxylate anion of 20:4 at *m/z* 303.233 and decarboxylate anion of 20:4 at *m/z* 259.241 was detected. The predicted structure of 20:4.

Based on the MS/MS fragmentation scheme, this molecular species was identified as lyso-glycerophosphoethanolamine 20:4 LPE.

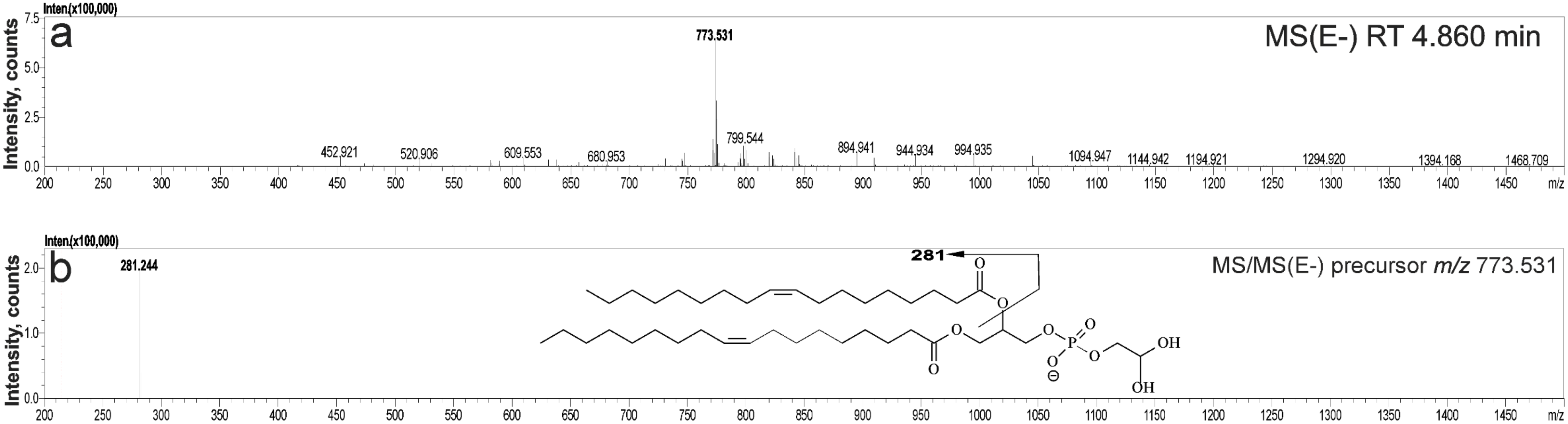

Fig. S6 Electrospray ionization mass spectra of proposed 18:1/18:1 PG. The total lipids of the HMC-3 were analyzed by high-performance liquid chromatography with tandem mass spectrometry (HPLC-MS/MS) in negative ion modes. **(a)** Mass spectrum for compounds eluting between 4.7-5.2 min. At tR = 4.860 min, the negative ion $[M–H]^-$ at *m/z* 773.531 was annotated as $[C_{42}H_{79}O_{10}P]^-$ (calculated 773.533). **(b)** MS/MS spectrum of the precursor ion at at *m/z* 773.531. The carboxylate anion of 18:1 at *m/z* 281.244 was detected. The predicted structure of 18:1/18:1.

Based on the MS/MS fragmentation scheme, this molecular species was identified as diacyl-phosphoglycerol 18:1/18:1 PG.

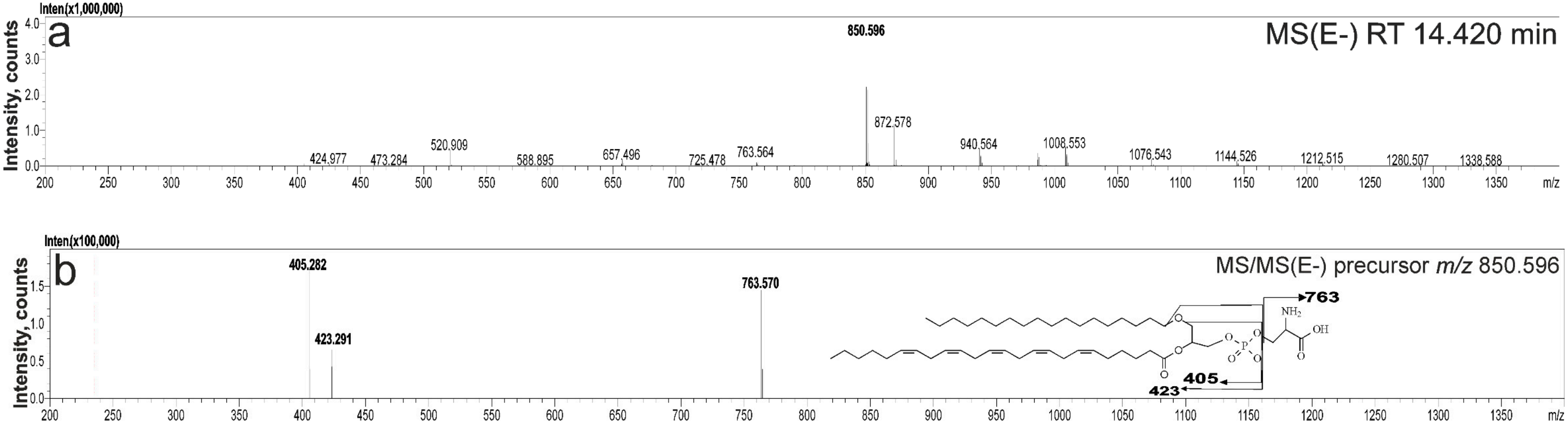

Fig. S7 Electrospray ionization mass spectra of proposed 18:0e/24:5 PS. The ePS fraction after column chromatography separation was analyzed by high-performance liquid chromatography with tandem mass spectrometry (HPLC-MS/MS) in negative ion modes. **(a)** Mass spectra for compounds eluting between 13.6-15.7 min. At tR = 14.420 min, the negative ion $[M–H]^{-}$ at *m/z* 850.596 was annotated as $[C_{48}H_{86}NO_9P]^{-}$ (calculated 850.596). **(b)** MS/MS spectrum of the precursor ion at *m/z* 850.596. Diagnostic fragments include the loss of 87 (serine head group) at *m/z* 763.570 and fragments at *m/z* 405.282 and *m/z* 423.291, confirming the 18:0 alkyl chain. The predicted structures of 18:0e/24:5.

Based on the MS/MS fragmentation scheme, this molecular species was identified as *O*-alkyl-acyl-glycerophosphoserine 18:0e/24:5 PS. The position of double bonds in the 24:5 acyl chain is assigned tentatively as n-6 based on literature data on octocoral fatty acid.

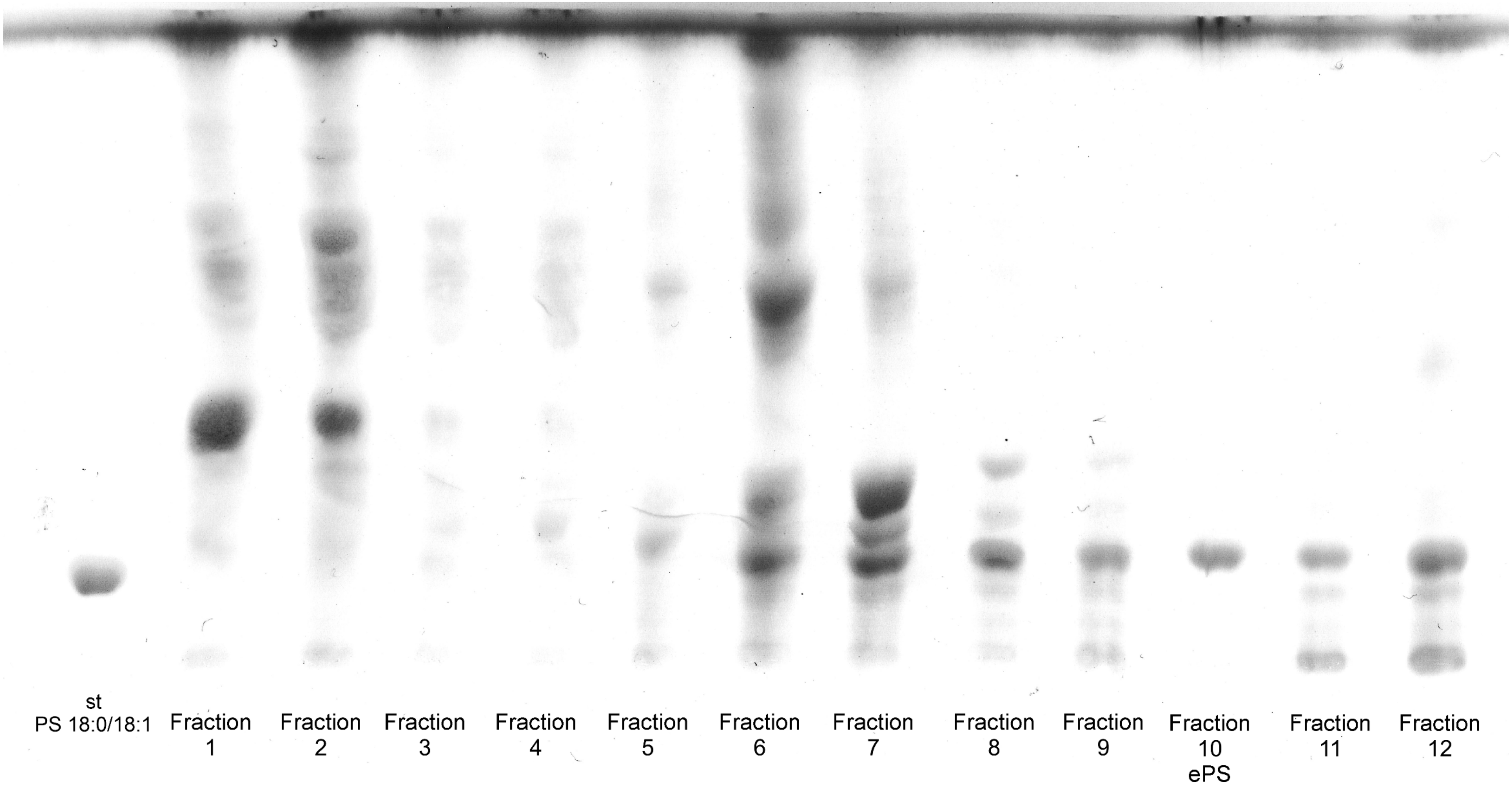

Fig. S8 Thin layer chromatography of the isolated 1-12 fractions and the standard PS 18:0/18:1. Fraction 10 contained ether phosphatidylserine (ePS) with PUFA 24:5.

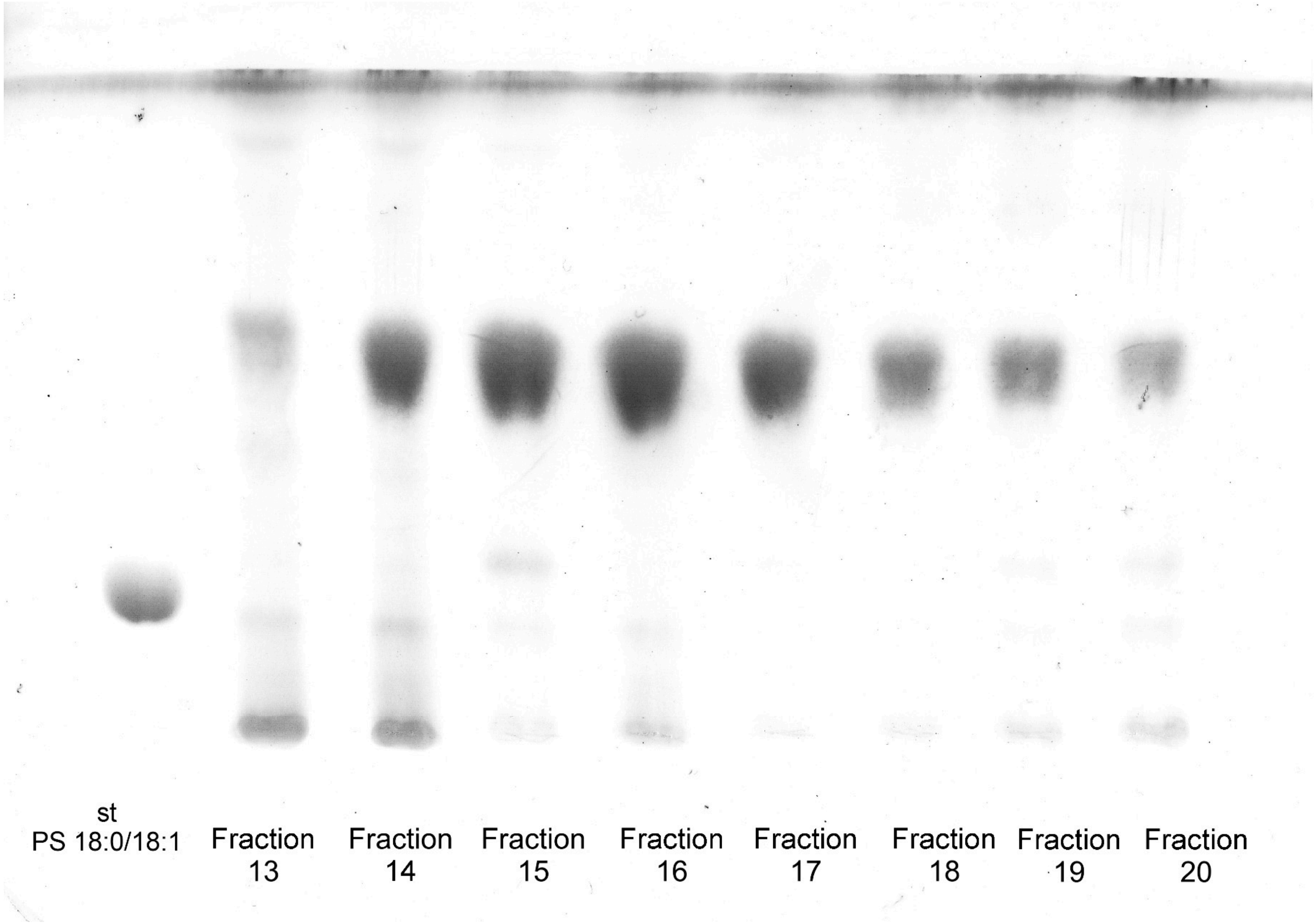

Fig. S9 Thin layer chromatography of the isolated 13-20 fractions and the standard PS 18:0/18:1.
